# Early Gut Fungal and Bacterial Microbiota and Childhood Growth

**DOI:** 10.3389/fped.2020.572538

**Published:** 2020-11-09

**Authors:** Kasper Schei, Melanie Rae Simpson, Ekaterina Avershina, Knut Rudi, Torbjørn Øien, Pétur Benedikt Júlíusson, David Underhill, Saideh Salamati, Rønnaug Astri Ødegård

**Affiliations:** ^1^Department of Clinical and Molecular Medicine, Faculty of Medicine and Health Sciences, NTNU – Norwegian University of Science and Technology, Trondheim, Norway; ^2^Department of Public Health and Nursing, Faculty of Medicine and Health Sciences, NTNU – Norwegian University of Science and Technology, Trondheim, Norway; ^3^Clinic of Laboratory Medicine, St. Olavs Hospital, Trondheim, Norway; ^4^Faculty of Chemistry, Biotechnology and Food Science, Norwegian University of Life Sciences, Ås, Norway; ^5^Department of Health Registries, Norwegian Institute of Public Health, Bergen, Norway; ^6^Department of Clinical Science, Faculty of Medicine, University of Bergen, Bergen, Norway; ^7^Division of Immunology Research, Cedars-Sinai, Los Angeles, CA, United States; ^8^Regional Centre of Obesity Research and Innovation (ObeCe), Trondheim University Hospital, Trondheim, Norway

**Keywords:** gut microbiota, mycobiota, fungi, childhood growth, height velocity

## Abstract

**Introduction:** Childhood growth is a sensitive marker of health. Animal studies show increased height and weight velocity in the presence of fungal as well as antibiotic supplement in feed. Human studies on early gut microbiota and anthropometrics have mainly focused on bacteria only and overweight, with diverging results. We thus aimed to investigate the associations between childhood growth [height and body mass index (BMI)] and early fungal and bacterial gut microbiota.

**Methods:** In a population-based cohort, a subset of 278 pregnant mothers was randomized to drink milk with or without probiotic bacteria during and after pregnancy. We obtained fecal samples in offspring at four time points between 0 and 2 years and anthropometric measurements 0 and 9 years. By quantitative PCR and 16S/ITS rRNA gene sequencing, children's gut microbiota abundance and diversity were analyzed against height standard deviation score (SDS) and BMI-SDS and presented as effect estimate (β) of linear mixed models.

**Results:** From 278 included children (149 girls), 1,015 fecal samples were collected. Maternal probiotic administration did not affect childhood growth, and the groups were pooled. Fungal abundance at 2 years was positively associated with height-SDS at 2–9 years (β = 0.11 height-SDS; 95% CI, 0.00, 0.22) but not with BMI-SDS. Also, higher fungal abundance at 1 year was associated with a lower BMI-SDS at 0–1 year (β = −0.09 BMI-SDS; 95% CI, −0.18, −0.00), and both bacterial abundance and bacterial alpha diversity at 1 year were associated with lower BMI-SDS at 0–1 year (β = −0.13 BMI-SDS; 95% CI, −0.22, −0.04; and β = −0.19 BMI-SDS; 95% CI, −0.39, −0.00, respectively).

**Conclusions:** In this prospective cohort following 0–9-year-old children, we observed that higher gut fungal abundances at 2 years were associated with taller children between 2 and 9 years. Also, higher gut fungal and bacterial abundances and higher gut bacterial diversity at 1 year were associated with lower BMI in the first year of life. The results may indicate interactions between early gut fungal microbiota and the human growth-regulating physiology, previously not reported.

**Clinical Trial Registration**: Clinicaltrials.gov, NCT00159523.

## Introduction

Childhood growth constitutes a prominent and important sign of bodily development, and thus this sensitive health marker is assured by growth control programs worldwide (1). Human growth comprises four overlapping phases including foetal, infancy, childhood, and pubertal growth. Each growth phase is driven by certain endocrine processes, as well as being influenced by genetic, nutritional, and environmental factors (1, 2). Recent investigations suggest that the gut microbiota could be a possible growth regulator too (3, 4).

The gut microbiota refers to the microbial community within our gastrointestinal tract, housing symbiotic microbes like bacteria and fungi. The fungal proportion of the microbiota is denoted mycobiota. Early gut microbiota patterns have been associated with childhood obesity with various findings (5–8), e.g., at 3 months the relative abundances of Firmicutes and Lachnospiraceae were positively and for *Bifidobacterium* spp. negatively associated with early overweight and obesity. Associations with height velocity have been poorly explored, but in pre-school children, height velocity has been found associated with certain Firmicutes spp. at 3 months and higher gut bacterial diversity at 0–3 years (4, 7, 9, 10). Since the 1950s, antibiotics have been widely used as growth promotors in livestock production (11). While early human antibiotic use may predispose for later childhood obesity (12), its possible effect on height velocity is less elucidated. A *Helicobacter pylori* eradication study with 1 week administering broad-spectrum antibiotics in 6–10-year-old children showed increased height standard deviation scores (SDS) by 20% in the intervention group compared with the control within 1 year, even when *H. pylori* was not eradicated (13). The same antibiotics-height association was observed in a large Finnish infancy cohort (12).

When the European Union banned the use of antimicrobials as growth promotors in animal production, the search for non-antimicrobial growth promotors in animals led to a wide-spread use of yeast and its cell wall products as new growth promotors (14). In the early human gut, the most abundant yeast genera are *Debaryomyces, Candida*, and *Saccharomyces*, with a development toward higher diversity of species (alpha diversity) and more *Saccharomyces cerevisiae* as the children age (15). In two randomized-controlled trials in which preterm neonates (28–32 and 30–37 weeks of gestational age, respectively) were supplemented with a probiotic *S. cerevisiae* strain (*Saccharomyces boulardii*), probiotic groups experienced greater weight gain than the control group (length was not measured in one study and increased non-significantly in the other) (16, 17). This indicates that the early mycobiota could promote early human growth. All the same, the possible role for mycobiota as a human growth promotor remains unexplored.

The objective of the current study was therefore to study associations between early gut fungal and bacterial microbiota and childhood height-SDS and BMI-SDS in a longitudinal cohort of healthy children up to the age of 9 years.

## Materials and Methods

### Materials

The aim of the current study was to investigate the association between early gut microbiota and childhood growth. The stool samples analyzed in this study were collected during a randomized trial of probiotics (ProPACT) (18). In total, 415 mothers were randomized to drink probiotic or placebo milk from inclusion to 3 months post partum. The probiotic milk contained 5 × 10^10^ colony-forming units (CFUs) of each of *Lactobacillus rhamnosus* GG and *Bifidobacterium animalis* subsp. *lactis* Bb-12 and 5 × 10^9^ CFUs of *Lactobacillus acidophilus* La-5, whereas the placebo milk was sterile and contained no probiotic bacteria. This maternal probiotic administration led to an increased presence and abundance of LGG in the infants' gut microbiotas at 10 days and 3 months, but no significant difference at 1 and 2 years, as previously shown (18). Apart from this, there were no other statistically significant differences in the microbiota composition or diversity between the groups (18). Since we considered these differences to be minimal, the two arms were pooled in the analysis of the present study.

In total, 278 of 415 participating children supplied 1,015 fecal samples at 10 days, 3 months, 1 and 2 years after birth (Table 1). The stool samples were collected from the diaper and transferred to a tube with 10 ml Cary-Blair transport medium (~20 times dilution) before immediate freezing at −18°C at home. The parents were instructed to collect one big spoon of fecal matter with an enclosed spoon as sampling equipment. After transport to the laboratory, the samples were stored at −80°C before further analyses. Self-reported questionnaires about the health and environment of the child were collected in pregnancy, 6 weeks after birth, at 1 and 2 years, with information on mode of delivery, breast-feeding length, antibiotic administration to mother and offspring, and gestational age.

**Table 1:** rRNA gene quantification and 16S/ITS rRNA gene region sequencing of fecal samples.

	**10 Days**	**3 Months**	**1 Year**	**2 Years**	**Total**
All fecal samples (count)	274	246	247	248	1,015
Detected bacterial DNA (16S rRNA gene region)	266 (97%)	243 (99%)	247 (100%)	243 (98%)	999 (98%)
Sequenced 16S rRNA V3–V4 gene region amplicons (after rarefaction)^a^	178 (65%)	193 (78%)	216 (87%)	170 (69%)	757 (75%)
Detected fungal DNA (ITS rRNA gene region)	153 (54%)	148 (60%)	163 (66%)	189 (76%)	653 (64%)
Sequenced ITS gene region amplicons (after rarefaction)^a^	15 (6%)	4 (2%)	7 (3%)	11 (4%)	37 (4%)

a *Samples were sequenced if the qPCR cycle threshold was <35 cycles to provide trustworthy results in the sequencing procedure. Few samples were excluded due to rarefaction*.

#### Ethics Approval and Consent to Participate

The parents signed an informed consent at inclusion and were once more informed when the anthropometry data were drawn, with the ability to withdraw, which two participants did. The study protocol was approved by the Regional Ethical Committee of Central Norway (2014/1796; Trial registration at Clinicaltrials.gov NCT00159523, registered 08.09.2005).

### Methods

#### Anthropometric Measurements

Height and weight were measured at routine follow-ups at public health centers. Height was measured supine <2 years and standing thereafter with a stadiometer, and weight was measured with a digital weight, according to Norwegian guidelines. Anthropometrics were collected and converted to SDS (z-scores) based on a large Norwegian child population reference (19). BMI-SDS constitutes a more explanatory and precise way to describe children's weight development since BMI-SDS is adjusted for age and sex. Likewise, height-SDS better presents the height growth and indicates along which height percentile curve the child grows. To identify data errors and outliers, we identified height-SDS and weight SDS values ≤ 3 and >3, as well as measurements where height decreased in two consecutive measurements. These growth curves were evaluated, and datapoints were removed when one could assume that the measurements were incorrectly recorded. To ensure good-quality anthropometric data before analysis, all individual growth curves were modeled for inspection.

#### Microbiota Analyses

The microbiota analyses are thoroughly explained in the Supplementary Material. Briefly, stool samples were homogenized before DNA was extracted using a bacterial protocol (20) as no fungal protocols for fecal DNA extraction were validated. However, although different extraction kits may produce differing total amounts of DNA, the relative proportions of various DNA abundances seem to largely correspond within the assays (21). We used bacteria-targeted primers (V3–V4 part of 16S rRNA gene) (22) and fungi-targeted primers (ITS1 part of 18S rRNA gene) (23) for quantification by quantitative PCR (qPCR). The qPCR cut-off value was set to the negative control if fungal abundance was lower than negative control, or excluded from analysis if cycle threshold value (CT value) at ≥45. CT values were converted to fungal and bacterial DNA concentrations using standard curves (Supplementary Material). Fungal quantification of the rRNA 18S/ITS1 gene region has been performed previously in bovine rumen studies (24), and recently, strongly correlated abundance estimations have been obtained using the ITS region (25). These qPCR quantifications of the microbial rRNA genes [16S (V3–V4) for bacteria and ITS1 for fungi] were therefore used as abundance markers in this study. The majority of bacterial samples were sequenced (Table 1). Ensuring high-quality sequencing, only fungal samples <35 CT were sequenced, hence only 37 fungal samples underwent sequencing. The 16S and ITS1 rRNA gene regions were sequenced with Illumina MiSeq and thereafter processed with the Quantitative Insights into Microbial Ecology pipeline and UPARSE for operational taxonomic unit (OTU) clustering, described previously (15, 20). Rarefaction cut-offs of 2,000 bacterial reads/sample and 6,000 fungal reads/sample were used to ensure even representation while retaining most samples. Taxonomic annotation of the OTUs were done against the Greengenes database v13.8 for bacteria, and using a self-curated concordance system for fungi, as there are no well-established methods for fungal annotation (15).

### Statistics

The influence of fungal and bacterial abundances and bacterial diversity on height-SDS and BMI-SDS was estimated using linear mixed models, accounting for repeated anthropometric measurements with individuals as random intercept and age as fixed slope and random slope in a maximum likelihood model. The distributions of bacterial and fungal abundances were right skewed and therefore log transformed. Abundances and diversity were tested against breastfeeding, length of breastfeeding, and delivery mode with linear mixed models. The models were tested for interaction between the abundance/diversity and age, which did not change the estimates and was therefore not included in the final model. The analyses were also controlled for use of antibiotics within 2 years without substantial effect on the associations; thus, unadjusted analyses are reported. Probiotic supplementation and antibiotic use were not associated with growth and are therefore not included. However, statistically significant associations were stratified into probiotic and placebo groups to ensure that the effect estimators for growth were consistent and to look for possible confounding by probiotics. The growth data were divided into three age groups for analysis: 0–1, 1–2, and 2–9 years. These analyses were computed in StataMP15 (StataCorp) and remained uncorrected due to their exploratory nature. Alpha diversity was measured in Shannon-index (*H*′), representing the individual microbial diversity and computed using PAST (26). Fungal diversity was only used as material description and not in the final analyses due to a low sample size. Fungal detection in samples were tested against antibiotic administration in children and length of breastfeeding and showed no significant differences. Correlation of consecutive samples was evaluated with Pearson's pair-wise correlation. The microbial calculations and heatmaps were conducted in R using PhyloSeq (27). The OTU analyses were conducted with ANCOM in R (28), with zero-prevalence cut-off at 0.9, corrected for multiple comparisons by the Benjamini-Hochberg procedure and dichotomised into high and low SDS for height and weight at 0 SDS. The significance level was set to α = 0.05.

## Results

### Study Population

From the 415 mother-child pairs in the ProPACT study, we included 278 participants with at least one childhood fecal sample and clinical follow-up data (67%). Included participants' health characteristics are compared with those without fecal samples (Table 2), showing that included mothers were 8 months older and more educated, and their offspring had received more antibiotics between the first and second years of life.

**Table 2:** Maternal and offspring characteristics.

**Participant characteristics**	**ProPACT participants with fecal samples (*n* = 278)**	**ProPACT participants without fecal samples (*n* = 136)**	***P*-value^**a**^**
Maternal age at delivery [mean (SD), years]	30.0 (4.3)	29.3 (4.8)	0.03
Cesarean sections [No. (%)]	35 (12.6)	–^b^	–^b^
Allocated to probiotics [No. (%)]	141 (50.5)	63 (43.6)	0.42
Maternal higher education [No. (%)]	217 (77.8)	79 (58.5)	<0.01
Female offspring [No. (%)]	149 (53.4)	57 (54.3)	0.88
Gestational age [mean (SD), weeks]	40.3 (1.57)	40.2 (1.68)	0.47
Birth weight [mean (SD), g]	3,633 (485)	3,617 (446)	0.78
Birth length mean (SD; cm)	50.5 (1.94)	51.5 (6.09)	0.18
Breastfed after 3 months [No. (%^c^)]	224 (97.4)	39 (97.5)	0.97
Formula fed after 3 months [No. (%^c^)]	98 (36.3)	18 (40.0)	0.63
Breastfed beyond 1 year [No. (%^c^)]	73 (28.1)	9 (29.0)	0.99
**CHILDREN RECEIVING ANTIBIOTIC TREATMENT WITHIN [NO. (%**^**C**^**)]**			
6 weeks	6 (2.5)	1 (1.5)	0.61
1 year	36 (13.9)	12 (14.3)	0.93
2 years	117 (41.9)	22 (25.9)	<0.01
Pregnant mothers receiving antibiotics [No. (%^c^)]	16 (6.5)	2 (2.9)	0.09
Overweight (BMI-SDS ≥1) at 7–9 years [No. (%^c^)]	44 (18.2)	–^b^	–^b^
Obesity (BMI-SDS ≥2) at 7–9 years [No. (%^c^)]	6 (2.5)	–^b^	–^b^

a *P-values calculated using χ^2^ test for binary variables and t-test for continuous variables*.

b *Not available*.

c *Percentage of total respondents of the present questionnaire*.

### Fungal and Bacterial Abundances and Diversities

**Figure 1:**
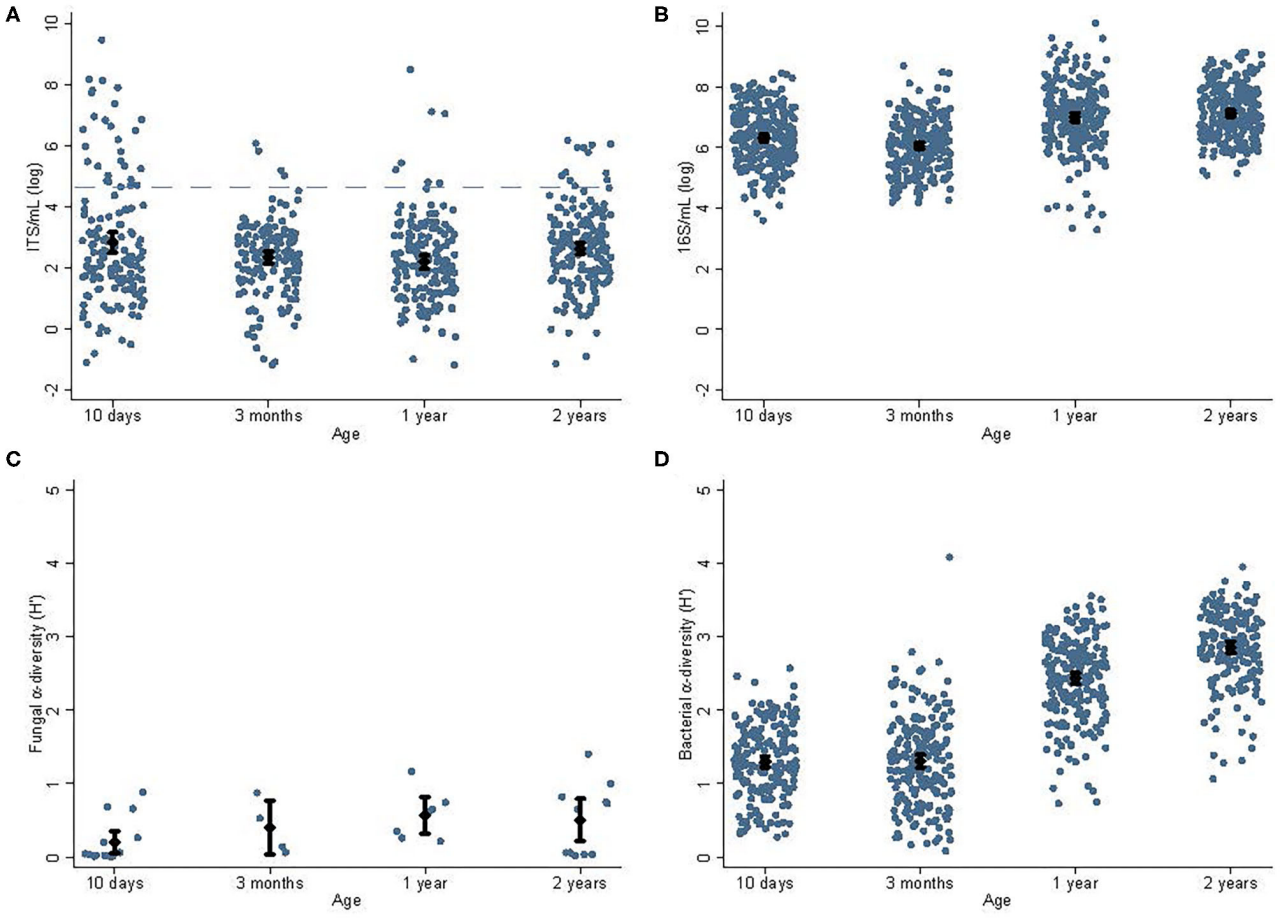
Abundances and alpha diversities for fungi and bacteria. Abundance and alpha diversity data for fungi and bacteria for children's samples at different ages [10 days, 3 months, 1, and 2 years; fungal data reported in (15)]. The average fungal abundances **(A)** decreased significantly (*P* = 0.01) from 10 days (2.83 log ITS/ml) to 1 year (2.19 log ITS/ml). The dashed blue line indicates the sequencing cut-off for fungi. Similarly, the bacterial abundance **(B)** decreased significantly (*P* = 0.04) from 10 days (6.31 log 16S/ml) to 3 months (6.06 log 16S/ml), and then increased toward 1 year (7.00 log 16S/ml, *P* < 0.01). There was insufficient data to determine the effect of age on the fungal alpha diversity **(C)**; however, bacterial alpha diversity **(D)** increased steadily from its lowest at 10 days (1.30 *H*′) and highest at 2 years (2.86 *H*′, *P* < 0.01). Cesarean section was associated with a non-significant trend toward lower bacterial alpha diversity at 3 months of age (1.09 vs. 1.36 *H*′, *P* = 0.06). Diamonds indicate sample means and error bars cover the 95% CI.

**Figure 2:**
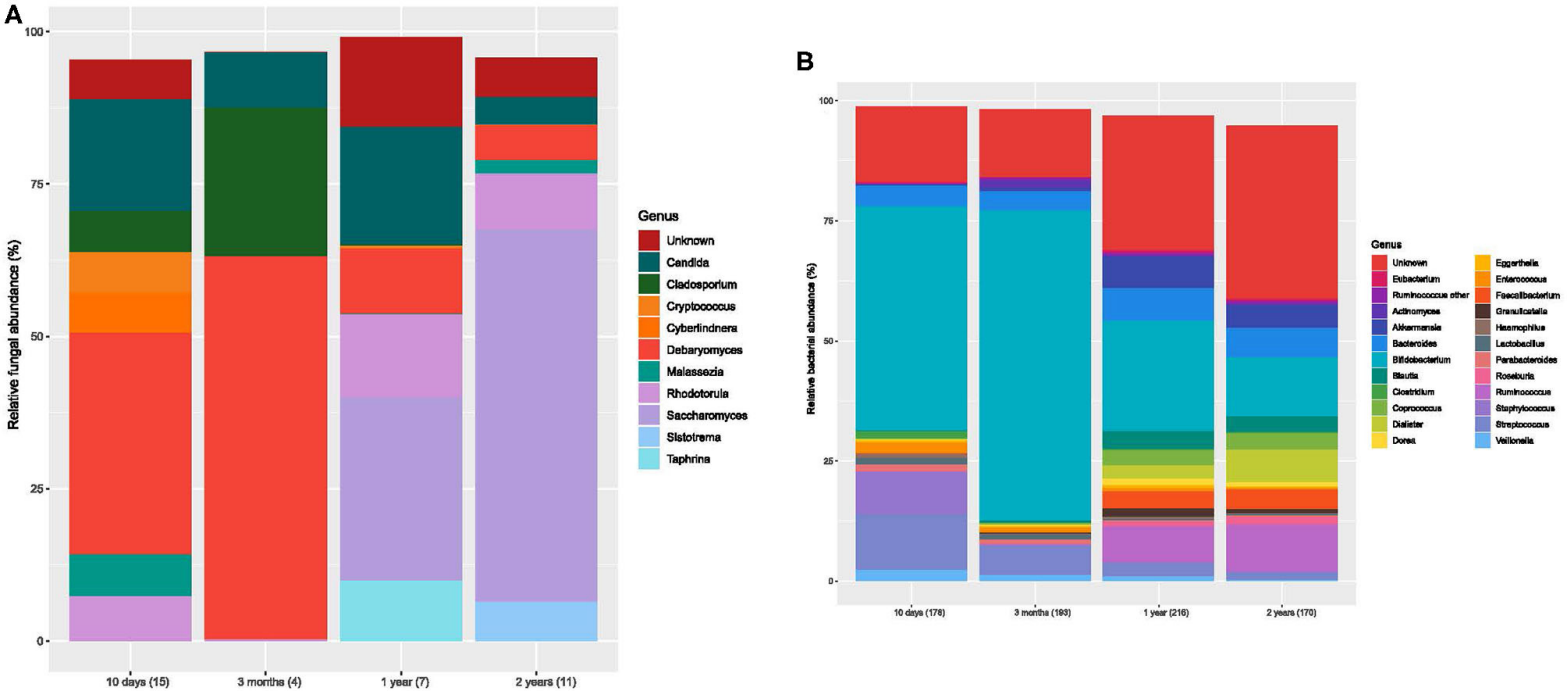
Bar charts of fungal and bacterial gut communities. **(A)** Mean relative abundances for the fungal genera (>1% abundant) for each age group. Each color designates a genus. Number of samples for each bar is stated in brackets below the bar. **(B)** Mean relative abundances for the bacterial genera for each age group. Each color designates a genera. The number of samples is stated in brackets below the bar.

The fungal and bacterial abundances and alpha diversities at different ages are shown in Figures 1, 2. The fungal data have been reported previously (15) but not in relation to bacterial data. There was no association between mode of delivery and fungal abundance, bacterial abundance, or bacterial alpha diversity in fecal samples collected from children; nor did antibiotic treatment within 6 weeks, 1, or 2 years of age correlate with fungal abundance, bacterial abundance, or bacterial alpha diversity (not shown). Duration of breastfeeding was not associated with fungal abundance, bacterial abundances, or bacterial alpha diversity in the mixed model analysis including all age groups. In a subgroup analysis, breastfeeding longer than 1 year was associated with lower bacterial diversity at 1 year −0.23 *H*′ (95% CI, −0.06 to −0.39), *P* = 0.007) but not at 2 years.

### Microbiota and Childhood Growth (Height-SDS and BMI-SDS)

About 13 (median; IQR, 12–16) data points for both weight and height per child were included in the analysis.

#### Zero- to One-Year Growth

**Figure 3:**
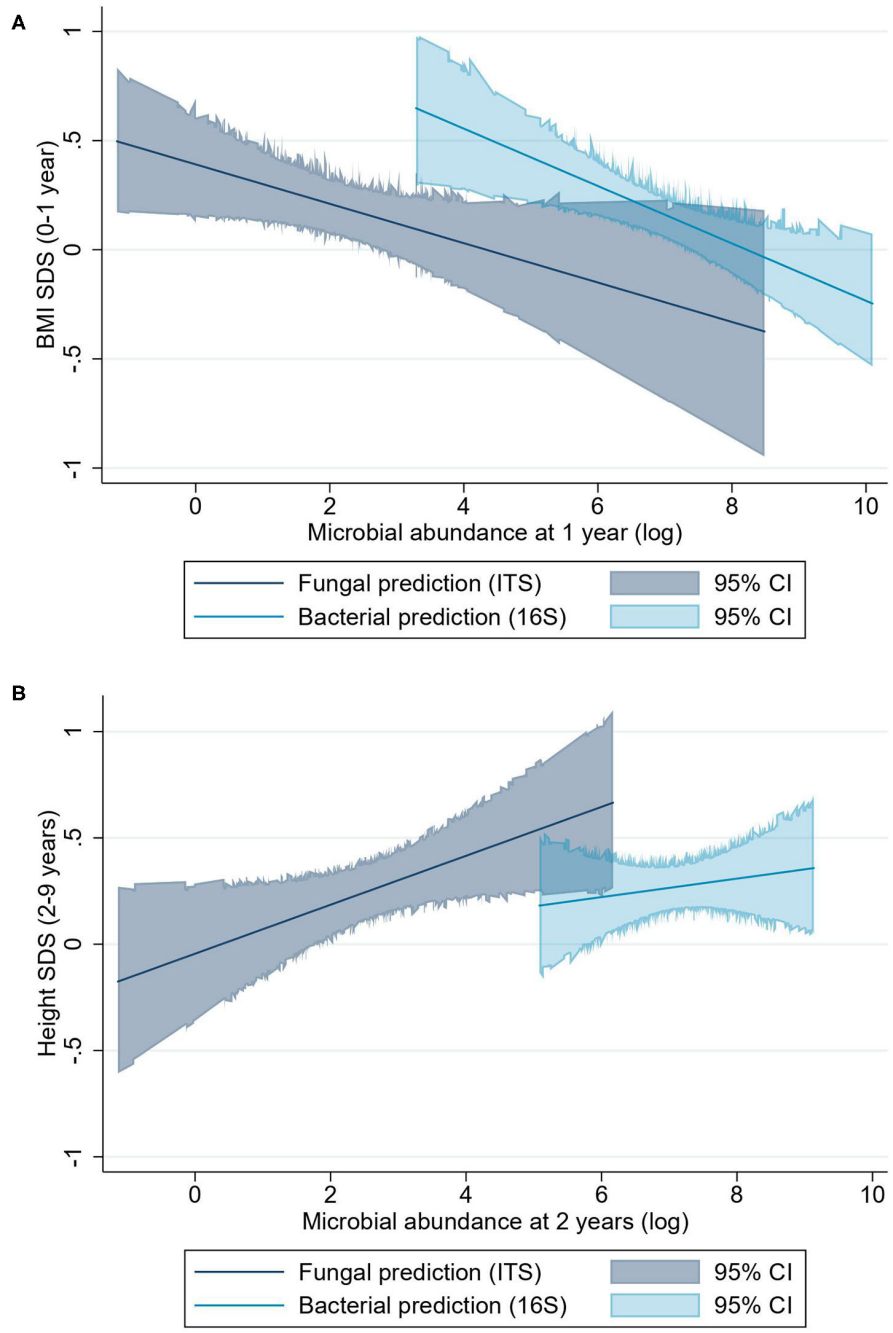
BMI-SDS and height-SDS and microbial abundance as predicted linear associations. **(A)** Predictions of BMI-SDS at 0–1 year for fungal and bacterial abundances. **(B)** Prediction of height-SDS at 2–9 years for fungal and bacterial abundance. The predictions are shown as lines, and the colored areas cover the 95% CI. The bacterial abundance prediction model for height-SDS remains statistically non-significant.

**Figure 4:**
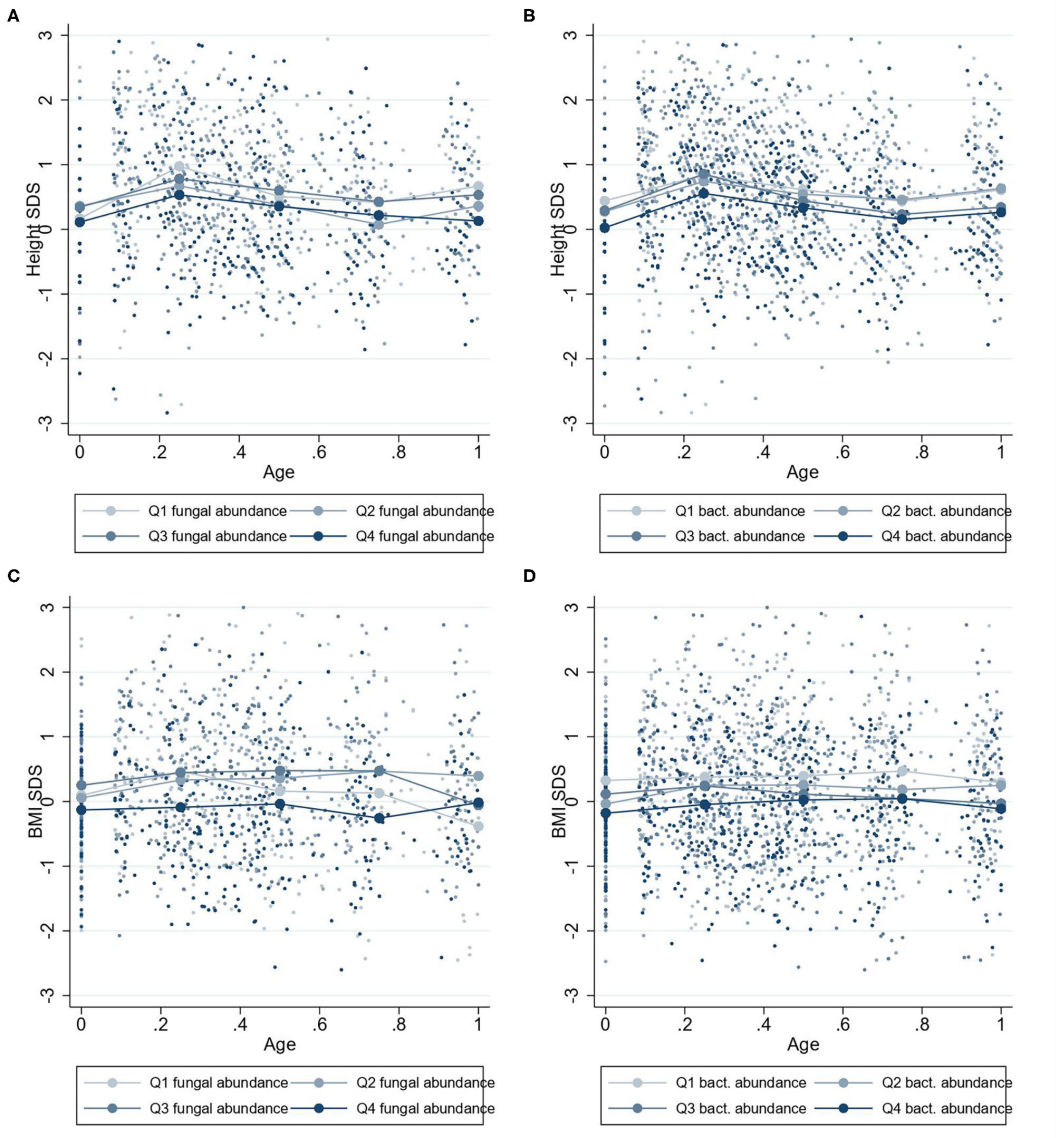
Mean height-SDS and BMI-SDS at 0–1 years according to microbiota abundances at 1 year. Mean standard deviation scores (SDS) values for children at 0–1 year with four quartiles of microbiota abundances at 1 year. Group mean height-SDS at 0–1 year for four quartiles of abundances of fungi **(A)** and bacteria **(B)**. Group mean BMI-SDS at 0–1 year for high or low abundances of fungi **(C)** and bacteria **(D)**.

The linear mixed regression model suggested that higher fungal abundance at 1 year was associated with a lower BMI-SDS from 0 to 1 year (β = −0.09 BMI-SDS; 95% CI, −0.18 to −0.00; *P* = 0.04) (Figure 3A). However, visualization of the relationship between fungal abundance quartiles and height-SDS indicates that this relationship may not be linear (Figure 4C). There was a trend that a higher fungal abundance in the 3-month sample also was associated to lower BMI-SDS at 0–1 year, but this did not reach statistical significance (β = −0.10 BMI-SDS; 95% CI, −0.20 to 0.00; *P* = 0.06). Bacterial abundance and bacterial alpha diversity at 1 year were also associated with lower BMI-SDS at 0–1 year (β = −0.13 BMI-SDS; 95% CI, −0.22 to −0.04; *P* = 0.004; and β = −0.19 BMI-SDS; 95% CI, −0.39 to −0.00; *P* = 0.047, respectively) (Figures 3A, 4, Supplementary Figure 1).

#### One- to Two-Year Growth

There were no statistically or clinically significant associations between fungal or bacterial abundances or bacterial diversity and height-SDS or BMI-SDS from 1 to 2 years (data not shown).

#### Two- to Nine-Year Growth

**Figure 5:**
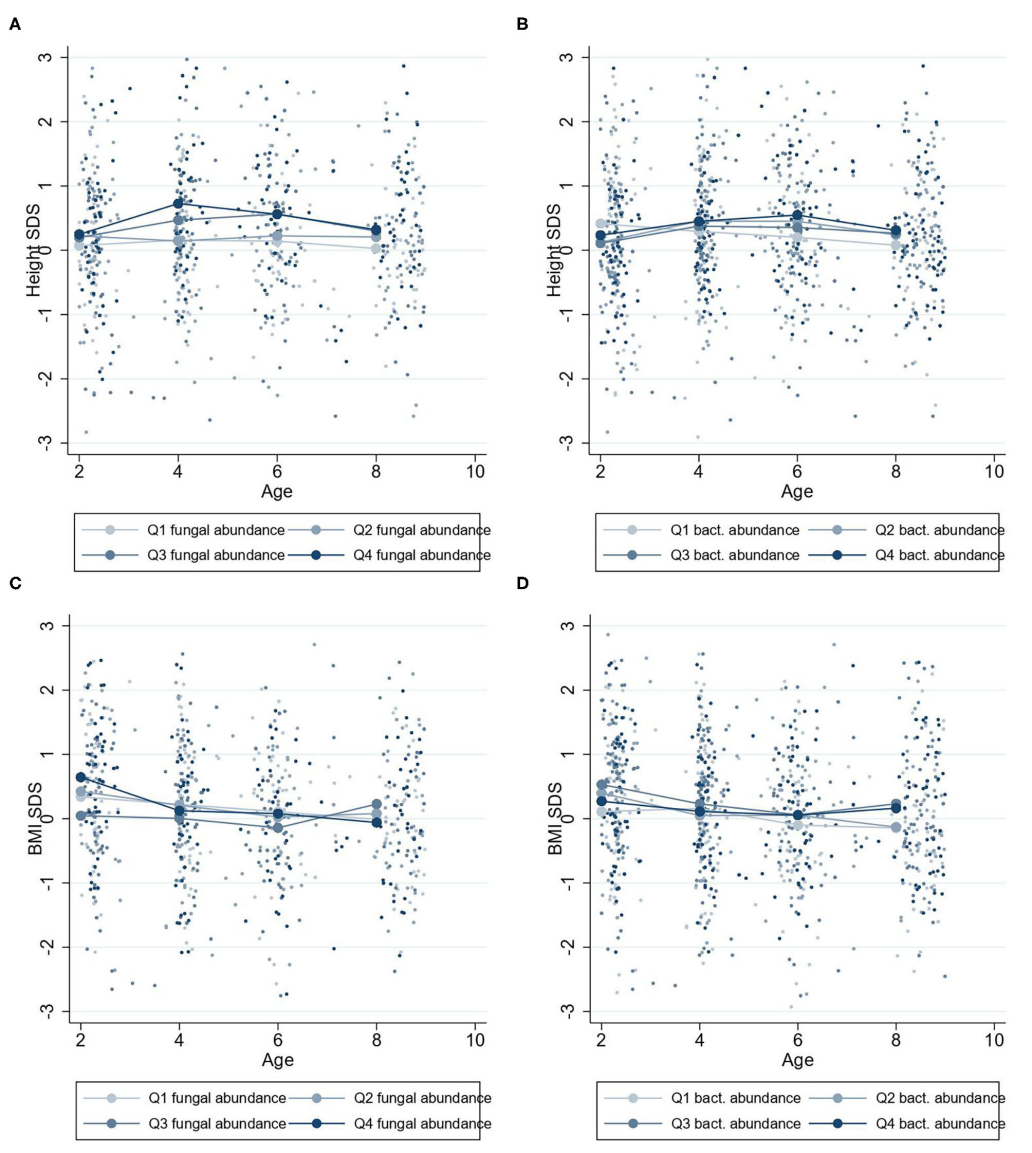
Mean height-SDS and BMI-SDS at 2–9 years according to microbiota abundances at 2 years. Mean standard curve deviation (SDS) values for children at 2–9 years with four quartiles of microbiota abundances at 2 years. Group mean height-SDS at 2–9 years for four quartiles of abundances of fungi **(A)** and bacteria **(B)**. Group mean BMI-SDS at 2–9 years for high or low abundances of fungi **(C)** and bacteria **(D)**.

Higher fungal abundance at 2 years was positively associated with height-SDS at 2–9 years (β = 0.11 height-SDS; 95% CI, 0.00–0.22; *P* = 0.04) (Figure 3B), and by visualization, the mean height-SDS was greater for each quartile of fungal abundance at all time points (Figure 5). There was no association with fungal abundance at 2 years and BMI-SDS at 2–9 years. Also, there was no association between bacterial abundance or bacterial alpha diversity and height-SDS or BMI-SDS (Figure 3, Supplementary Figure 1).

#### Microbial Taxa

Neither height-SDS nor BMI-SDS appeared to be associated with compositions of microbial communities (Supplementary Figure 2). For longitudinal ANCOM models analysing individual taxa, no individual taxa were associated with anthropometry. For ANCOM models including fecal samples at 2 years and anthropometry from 2 to 9 years, there was a negative association between relative abundance of *Bifidobacterium longum* and height-SDS (Supplementary Figure 3). No other microbial taxa differed significantly with height-SDS or BMI-SDS, indicating that the taxa abundances stay relatively stable with increased total abundance (at least for bacteria).

## Discussion

In this prospective population study, we found that greater abundances of gut mycobiota at 2 years were associated with increased height in children at 2–9 years. Furthermore, greater fungal and bacterial abundance and greater bacterial diversity at 1 year of age were associated with lower BMI-SDS in children in the first year of life. These new findings may suggest a link between the gut microbiota and childhood growth.

A greater fungal abundance in the 2-year fecal samples was associated with taller children from 2 to 9 years and was supported by an increasing trend in height-SDS in the quartile analysis (Figure 5A). Assuming the range of fungal abundance of 6 units at 2 years (Figure 1), this would represent a difference of about 3–4 cm at 6 years of age. This finding was in accordance with our hypothesis that a more abundant mycobiota could affect future height. Growth stimulation by adding *S. cerevisiae* into the feed has been shown in piglets and dairy cows, possibly through the growth hormone (GH) axis (29, 30). *S. cerevisiae* is one of many fungi found in the human gut mycobiota, with increasing abundance toward 2 years of age (15). The GH axis becomes the driving growth regulator from 1 to 2 years when entering the childhood growth phase (1, 2), which may justify why the association between fungal abundance and height growth becomes apparent from 2 years of age.

Children hosting higher abundances of fungi and bacteria and higher bacterial alpha diversity at 1 year had lower BMI in their first year of life, in this cohort of healthy well-nourished Norwegian children with BMI-SDS normally distributed around zero. Assuming the same range of microbial abundance of 6 units at 1 year (Figure 1), this would represent a BMI difference of about 1 BMI unit at 1 year of age. We also observed a tendency that higher fungal abundance at 3 months correlated with lower BMI-SDS at 0–1 years. Thus, the relation between BMI-SDS and microbial abundance and bacterial diversity depicts a process happening after the first months of life. Our data do not prove a causal direction in the analysis of microbial abundances/diversity and infantile BMI. However, the indication at 3 months could suggest that at least fungal abundance increases at least within a few months after birth in those with lower infantile BMI-SDS. The gut microbial abundance and diversity normally increase from birth to 1 year (31), and having a considerably high microbial diversity and abundance as food is introduced might be favorable for a lower BMI development. High bacterial diversity has been associated with childhood and adult leanness (32), in accordance with our finding.

Interestingly, the taxonomic analysis yielded no associations with BMI-SDS, using established and conservative methods. This is in contrast with several recent investigations that showed divergent associations with BMI and microbes (5–8). The lack of consistent findings could be due to sample variations, liberal statistical tests, or varying methods. By investigating microbial total abundance, we observed links to both height velocity and lower BMI. The absolute abundances appear thus to reveal more than the microbial composition concerning growth. More rigorous methods and statistical tools in this research field are required (and are under development) and will hopefully provide more robust analyses in the future.

We found no associations between antibiotic usage and growth. This contrasts other human studies showing increased childhood longitudinal growth after broad-spectrum antibiotics treatments (12, 13). The livestock growth promotors are low doses of broad-spectrum antibiotics continually, whereas the children in our cohort received short-time treatments of narrow-spectrum antibiotics. Thus, the different treatment lengths and varying antimicrobial spectrums may explain the differing findings.

This large population-based cohort of healthy Norwegian children has a 9-year-long follow-up that enabled us to explore associations between childhood growth and gut microbiota. A conservative OTU approach decreased the rate of type I error findings, and the bacterial analysis is robust. We managed to quantify fungal DNA abundances in most samples, although the lack of well-established fungal DNA extraction protocols validated for stools might have reduced the extraction rate of fungal DNA. Underlining the difficulty of fungal analyses, low fungal amounts in general and a bacterially focused DNA extraction made us unable to describe the total fungal diversity as only 37 samples were sequenced for fungi, although 64% of samples were quantified to measure microbial abundances. Furthermore, the parents collected the fecal samples, which could represent a random sampling misclassification. There are no databases for the number of repeats of fungal ribosomal RNA operons (*rrn*) for every fungal species detected, which could impair the quantification precision. Also, as for all DNA-based microbiome sequencing studies, the proportion of inactive transient microbes remains unknown. Therefore, these findings should be replicated, preferably with fungal-specific extraction kits. However, this is the first study to show an association between childhood growth and early gut mycobiota abundance, introducing a novel research area on how early gut mycobiota may impact human health and might possibly serve as a growth promotion target.

## Conclusion

In a 9-year follow-up of healthy well-nourished children, increased gut fungal abundance appears to be more strongly associated with childhood anthropometrics (increased height velocity and reduced BMI) than bacterial abundance and diversity (reduced BMI only). Analysing gut fungi remains challenging; nevertheless, the findings call for more research on how the mycobiota could affect human growth physiology.

## Data Availability Statement

The data analyzed in this study is subject to the following licenses/restrictions: The datasets of the current study are not publicly available due to legislation of the Norwegian Authorities on the sharing of personal yet non-identifiable data. The datasets are available upon reasonable request and are stored at our university. Requests to access these datasets should be directed to Torbjørn Øien, torbjorn.oien@ntnu.no.

## Ethics Statement

The studies involving human participants were reviewed and approved by Regional Ethical Committee of Central Norway (2014/1796). Written informed consent to participate in this study was provided by the participants' legal guardian/next of kin.

## Author Contributions

KS was involved in the microbiome data generation, did the statistical analysis, interpretation, and drafted the initial manuscript. MS and PJ contributed to the statistical analysis and interpretation and reviewed and revised the manuscript. EA and KR were involved in the microbiome data generation and reviewed and revised the manuscript. TØ designed the study, enrolled the participants, coordinated and supervised the data collection, and reviewed and revised the manuscript. DU and SS were involved in the conceptualisation and design of the study and reviewed and revised the manuscript. RØ supervised the study, conceptualized and designed the study, interpreted the data, and reviewed and revised the manuscript. All authors approved the final manuscript as submitted and agreed to be accountable for all aspects of the work. All authors contributed to the article and approved the submitted version.

## Conflict of Interest

The authors declare that the research was conducted in the absence of any commercial or financial relationships that could be construed as a potential conflict of interest.

## Abbreviations

Bb-12: *Bifidobacterium animalis* subsp. *lactis* Bb-12
BMI: body mass index
CFU: colony-forming unit
CI: confidence interval
CT: Cycle threshold
ITS: internal transcribed spacer
GH: growth hormone
La-5: *Lactobacillus acidophilus* La-5
LGG: *Lactobacillus rhamnosus* GG
OTU: operational taxonomic unit
qPCR: quantitative polymerase chain reaction
QIIME: Quantitative Insights into Microbial Ecology
*rrn*: ribosomal RNA operons
SDS: standard deviation score
sp./spp.: species (singular/plural).
