# Supplementary material for: Early Gut Fungal and Bacterial Microbiota and Childhood Growth
Source: Front Pediatr. 2020 Nov 9;8:572538. doi: 10.3389/fped.2020.572538 (PMC7680752; doi:10.3389/fped.2020.572538)
Supplement: Supplementary file 1 [file Data_Sheet_1.docx]

***Supplementary Material***

- Supplementary Methods
- Supplementary Table 1
- Supplementary Table 2
- Supplementary Figure 1
- Supplementary Figure 2
- Supplementary Figure 3
- Standard curve calculations
- Supplementary References

**Supplementary Data**

We used a protocol for bacterial DNA extraction that involved mechanical and chemical cell lysis. Stool samples were first homogenised by bead-beating with acid-washed glass beads (Sigma, USA), before DNA was extracted by LGC Mag DNA extraction kit (LGC Genomics, Middlesex, UK),^1^ following the manufacturer’s instructions, including a negative control as contamination control.

We used different primers for bacteria and fungi to quantify their abundances with quantitative PCR (qPCR). The V3-V4 part of the bacterial 16S ribosomal RNA (rRNA) gene region was quantified using the primer pair targeting prokaryotic organisms (PRK341F (CCTACGGGRBGCASCAG) and PRK806R (GGACTACYVGGGTATCTAAT))^2^, with a LightCycler qPCR (Roche, Switzerland). Thermocycles comprised 95 °C in 15 min, then (95 °C for 30 s, 55 °C for 30 s, 72 °C for 45 s) × 40. For each qPCR plate, we included positive and negative controls (*Escherichia coli* and sterile water, respectively). The internal transcribed spacer 1 (ITS1) of the fungal 18S rRNA gene region was quantified using the primer pairs targeting fungi (ITS1F (CTTGGTCATTTAGAGGAAGTA) and ITS2 (GCTGCGTTCTTCATCGATGC)),^3^ with a LightCycler qPCR. Fungal quantification of the rRNA 18S/ITS1 gene region has been performed previously in bovine rumen studies,^4^ and recently, strongly correlated abundance estimations have been obtained using the ITS region.^5^ Thermocycles comprised 95 °C in 15 min, then (95 °C in 30 s, 56 °C in 30 s, 72 °C for 45 s) × 50. For each qPCR plate, we included positive and negative controls (*S. cerevisiae* and sterile water, respectively). The qPCR cycle threshold (CT) value cut-off for fungal detection was set to either within the value of the negative control or to 45 cycles, because our experience suggests that DNA quantification beyond 45 cycles may produce misleading results. The qPCR CT values were converted to fungal and bacterial DNA concentration using standard curves for CT values with measured DNA concentrations (Online Text).

We sequenced the bacterial 16S rRNA gene region amplicons with Illumina MiSeq in three runs (Supplementary Table 1), previously described in detail.^1^ We also sequenced a selection of the fungal ITS1 rRNA gene region amplicons with Illumina MiSeq, previously described in details.^6^ We used a CT value cut-off of 35 to ensure high-quality of the sequencing and thus only 37 fungal samples underwent sequencing. We used the Quantitative Insights into Microbial Ecology (QIIME) pipeline for quality filtering and the UPARSE algorithm for operational taxonomic unit (OTU) clustering for both bacterial and fungal sequencing results.^7^ To ensure even representation and to retain as many samples as possible, we applied a rarefaction cut-off of 2000 reads per sample for the bacterial data, whereas a cut-off of 6000 reads per sample was deemed to be most appropriate for the ITS data, as described previously.^1,6^ Only three of the participating children had repeated sequencing results, all three at 10 days and 2 years.

The fungal sequencing created a total of 1 576 611 quality-filtered reads (median 16 367, minimum 3, maximum 119 463), and 21 018 190 quality-filtered reads (median 7 172, minimum 10, maximum 169 024) were rendered from the bacterial sequencing (age-specific numbers in Supplementary Table 1). The fungal and bacterial communities showed a succession from birth towards a more adult-like gut microbiota composition at 2 years, as reported previously from the ProPACT study.^1,6^

The bacterial OTUs were taxonomically annotated against the Greengenes database v13.8. Since there is no well-established method for mycobiotic taxonomy assignment, we used a conservative concordance system in which we compared four taxonomical databases (GenBank, Warcup Fungal ITS, UNITE fungal ITS and Targeted Host-Associated Fungi ITS Database) and assigned the species from the agreement of these databases.^6^

**Supplementary Table 1A. Quality-filtered fungal reads.**

|  | Total reads | median | min | max |
| --- | --- | --- | --- | --- |
| 10 days | 994912 | 17007 | 3 | 119463 |
| 3 months | 143928 | 33040.5 | 22633 | 55214 |
| 1 year | 211787 | 30980 | 8378 | 49377 |
| 2 years | 225984 | 12694 | 1509 | 85140 |

**Supplementary Table 1B. Quality-filtered bacterial reads.**

|  | Total reads | median | min | max |
| --- | --- | --- | --- | --- |
| 10 days | 3002586 | 3932 | 24 | 164269 |
| 3 months | 3486014 | 8385 | 72 | 96170 |
| 1 year | 4205606 | 10231.5 | 15 | 169024 |
| 2 years | 2677290 | 4690 | 21 | 158031 |

**Supplementary Table 2A. Associations between fungal abundance (ITS) and anthropometry**

|  | **Height-SDS**^1^ | **BMI-SDS**^1^ |
| --- | --- | --- |
| 10 days | | |
| - 1. yr (beta) (95% CI)   (p-value) | -0.034 (-0.37) -0.10, 0.03 0.28 | -0.01 (-0.02) -0.08, 0.05 0.65 |
| - 1. yr (beta) (95% CI)   (p-value) | 0.01 (0.00) -0.07, 0.08 0.88 | 0.00 (0.00) -0.07, 0.08  0.94 |
| - 1. yr (beta) (95% CI)   (p-value) | -0.03 (-0.03)  -0.10, 0.04  0.35 | 0.01 (0.01)  -0.06, 0.08  0.81 |
| 3 months | | |
| - 1. yr (beta) (95% CI)   (p-value) | 0.04 (0.05)  -0.06, 0.15 0.43 | **-0.10 (-0.09) -0.20, 0.00**  **0.06** |
| - 1. yr (beta) 95% CI)   (p-value) | -0.03 (-0.04)  -0.17, 0.10  0.62 | -0.01 (-0.01)  -0.14, 0.12  0.88 |
| - 1. yr (beta) 95% CI)   (p-value) | 0.03 (0.03)  -0.10, 0.15  0.68 | 0.03 (0.03)  -0.10, 0.16  0.64 |
| 1 year | | |
| - 1. yr (beta)   (95% CI)  (p-value) | -0.07 (-0.07) -0.16, 0.03 0.14 | **-0.09 (-0.08)**  **-0.18, 0.00**  **0.044** |
| - 1. yr (beta) (95% CI)   (p-value) | -0.04 (-0.04)  -0.14, 0.07  0.48 | -0.03 (-0.03)  -0.15, 0.08  0.58 |
| - 1. yr (beta) (95% CI)   (p-value) | -0.02 (-0.02)  -0.12, 0.09  0.74 | -0.03 (-0.02)  -0.13, 0.07  0.59 |
| 2 years | | |
| - 1. yr (beta) (95% CI)   (p-value) | 0.07 (0.07)  -0.02, 0.16  0.15 | 0.03 (0.02)  -0.06, 0.11  0.57 |
| - 1. yr (beta) (95% CI)   (p-value) | 0.07 (0.06)  -0.04, 0.18  0.23 | -0.02 (-0.02)  -0.13, 0.10  0.79 |
| - 1. yr (beta) (95% CI)   (p-value) | **0.11 (0.11)**  **0.00, 0.22**  **0.041** | 0.03 (0.03)  -0.07, 0.14  0.53 |

^1^Corrected beta value within the parenthesis (corrected for whether the mother received probiotic supplementation or not).

**Supplementary Table 2B. Associations between bacterial abundance (16S) and anthropometry**

|  | **Height-SDS**^1^ | **BMI-SDS**^1^ |
| --- | --- | --- |
| 10 days | | |
| - 1. yr (beta)   (95% CI)  (p-value) | -0.06 (-0.06)  -0.17, 0.05  0.30 | 0.02 (0.02)  -0.09, 0.12  0.72 |
| - 1. yr (beta) (95% CI)   (p-value) | -0.06 (-0.05)  -0.19, 0.08  0.42 | 0.07 (0.06)  -0.07, 0.20  0.33 |
| - 1. yr (beta) (95% CI)   (p-value) | 0.04 (0.04)  -0.09, 0.17  0.53 | -0.00 (-0.00)  -0.13, 0.13  0.97 |
| 3 months | | |
| - 1. yr (beta)   (95% CI)  (p-value) | -0.03 (-0.02)  -0.15, 0.08  0.58 | 0.02 (0.02)  -0.10, 0.13  0.78 |
| - 1. yr (beta) 95% CI)   (p-value) | -0.05 (-0.04)  -0.19, 0.10  0.51 | 0.10 (0.10)  -0.05, 0.25  0.18 |
| - 1. yr (beta) 95% CI)   (p-value) | -0.04 (-0.03)  -0.18, 0.10  0.59 | 0.03 (0.04)  -0.10, 0.17  0.63 |
| 1 year | | |
| 0-1 yr (beta)  (95% CI)  (p-value) | -0.08 (-0.09=  -0.18, 0.01  0.08 | **-0.13 (-0.13)**  **-0.22, -0.04**  **0.004** |
| - 1. yr (beta) (95% CI)   (p-value) | -0.06 (-0.06)  -0.17, 0.06  0.29 | -0.07 (-0.07)  -0.19, 0.05  0.25 |
| - 1. yr (beta) (95% CI)   (p-value) | -0.07 (-0.07)  -0.18, 0.04  0.21 | -0.07 (-0.07)  -0.18, 0.04  0.24 |
| 2 years | | |
| - 1. yr (beta)   (95% CI)  (p-value) | 0.00 (0.00)  -0.12, 0.13  0.94 | -0.05 (-0.05)  -0.17, 0.07  0.41 |
| - 1. yr (beta) (95% CI)   (p-value) | -0.01 (-0.01)  -0.16, 0.14  0.87 | 0.02 (-0.02)  -0.13, 0.18  0.76 |
| - 1. yr (beta) (95% CI)   (p-value) | 0.04 (0.04)  -0.10, 0.19  0.56 | 0.09 (0.09)  -0.06, 0.23  0.24 |

^1^ Corrected beta value within the parenthesis (corrected for whether the mother received probiotic supplementation or not).

**Supplementary Figure 1.**


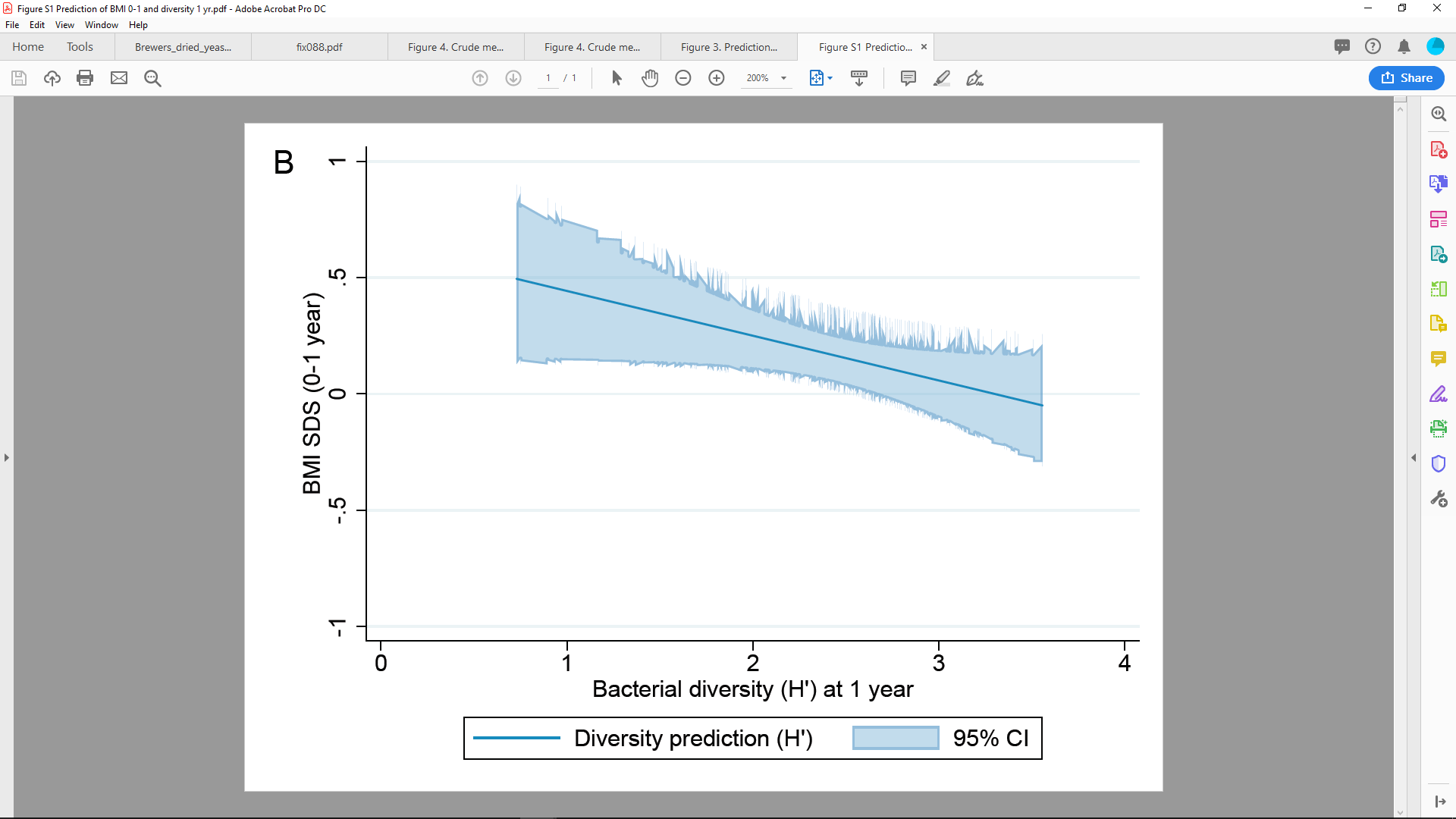


Supplementary Figure 1. BMI SDS at 0-1 year and bacterial diversity (Shannon index, H’) at 1 year as predicted linear association.


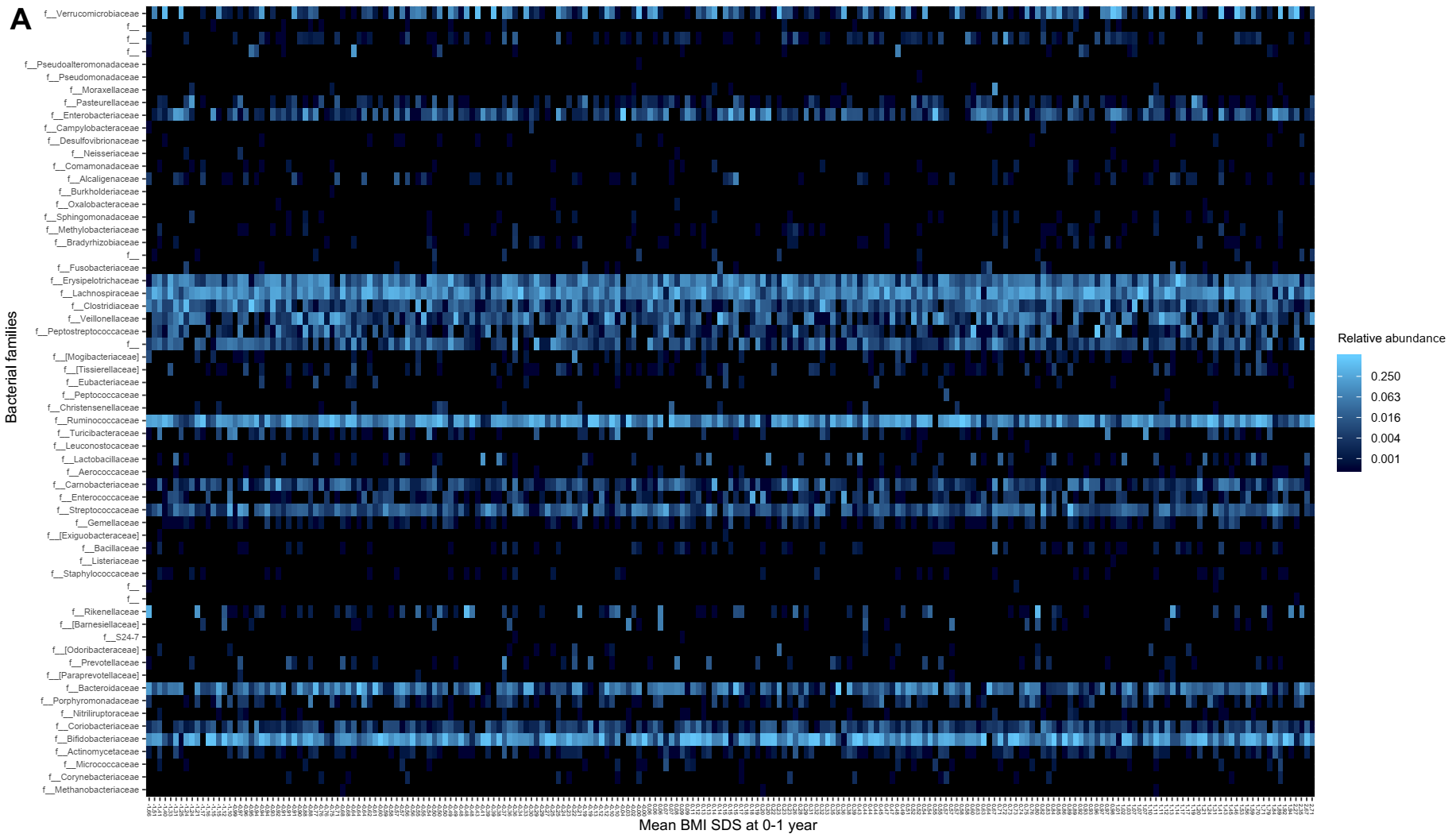
**Supplementary Figure 2. Heat maps of fungal and bacterial gut microbiota and anthropometric measurements.**


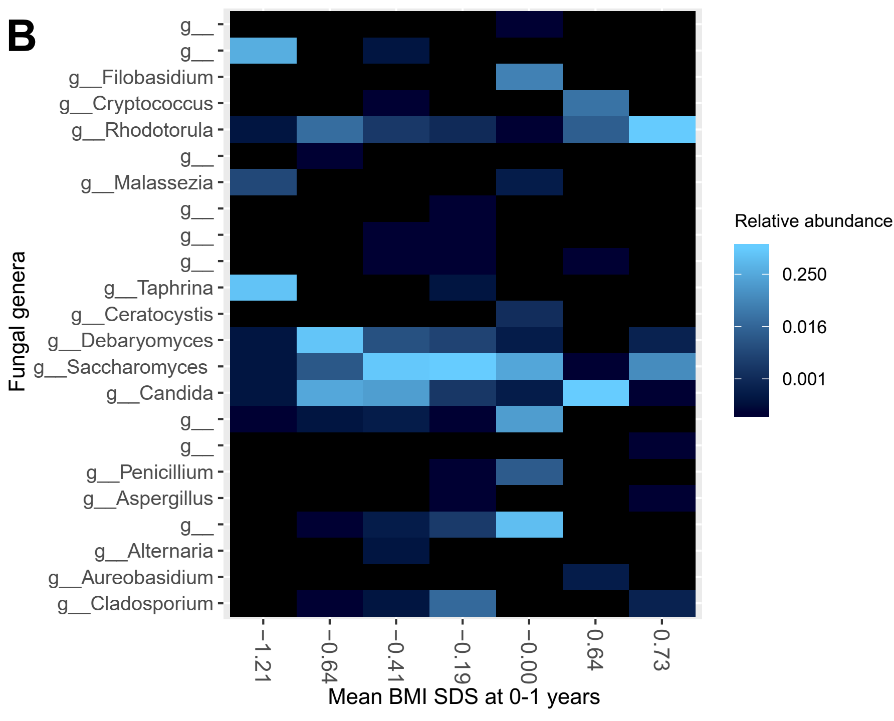


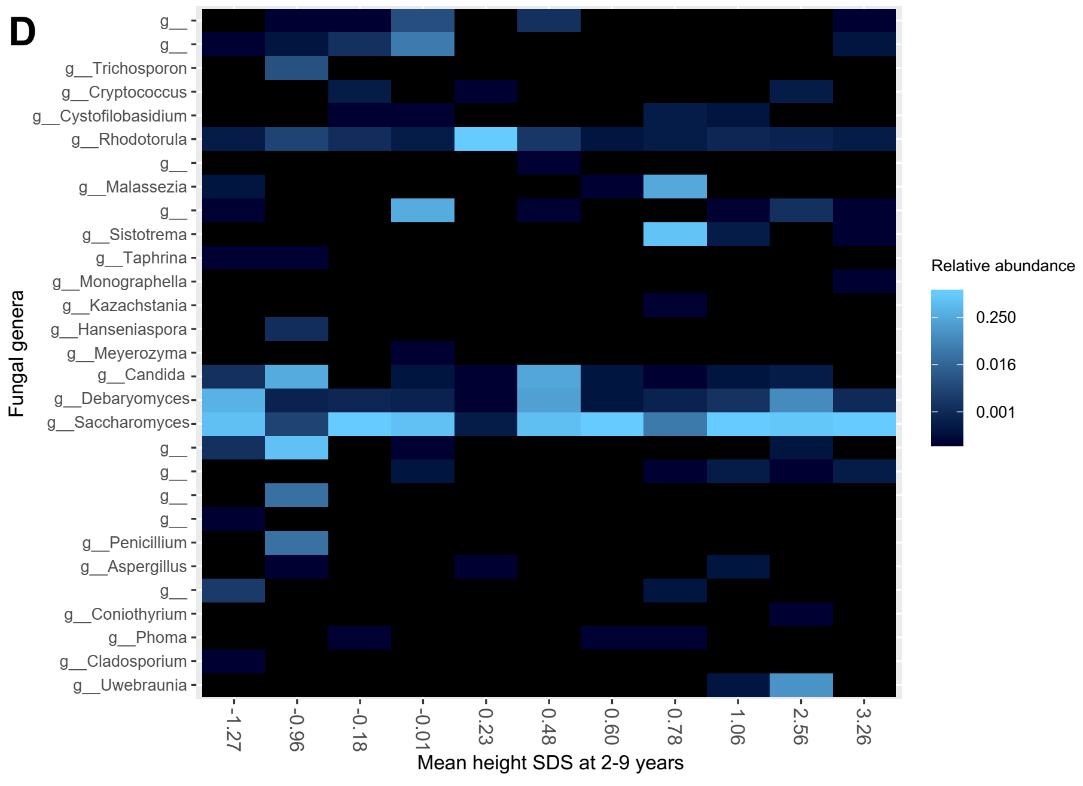

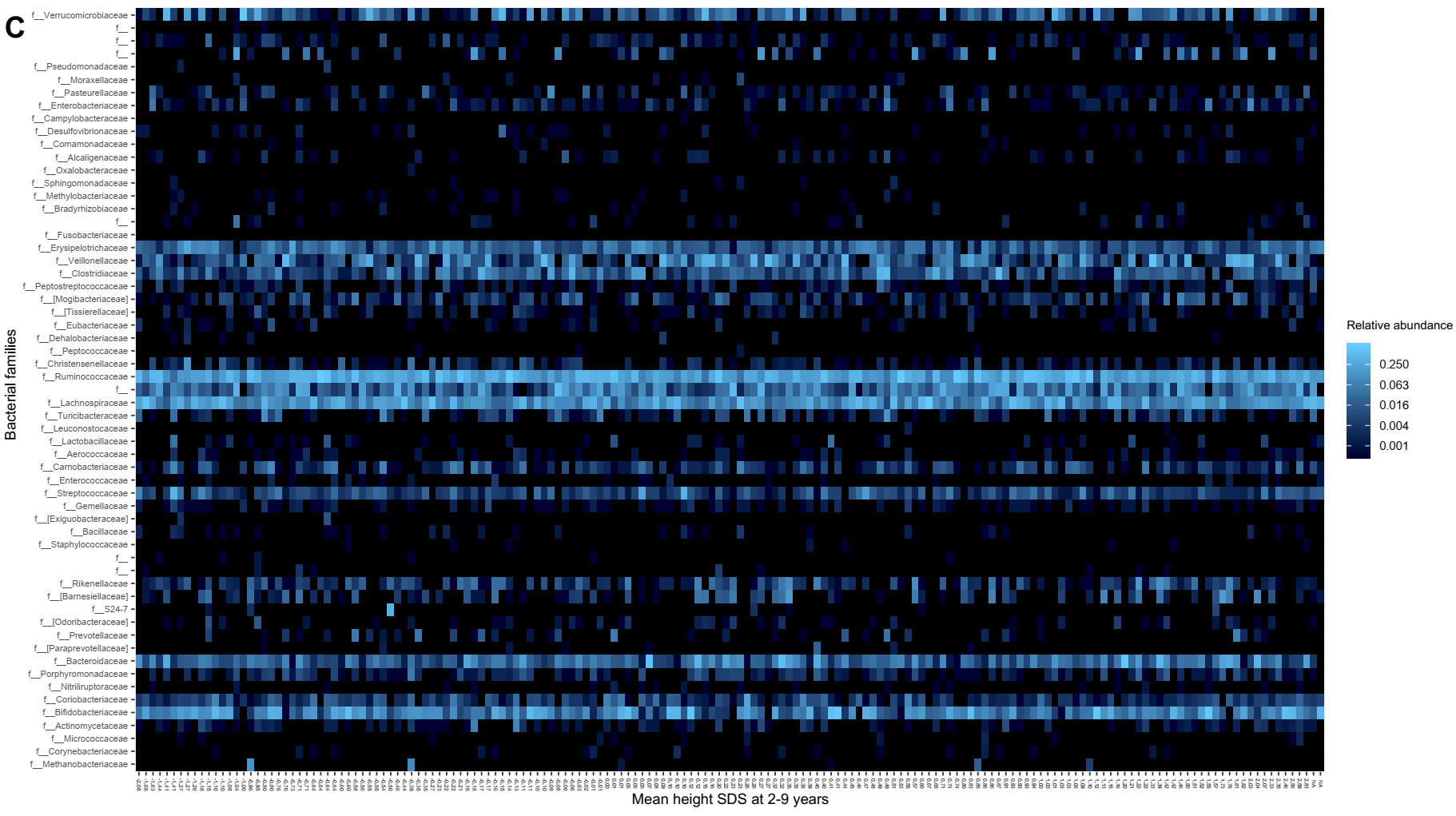


A, 1-year samples of bacterial community and BMI SDS from 0-1 years of age.
B, 1-year samples of fungal community and BMI SDS from 0-1 years of age.
C, 2-year samples of bacterial community and height SDS at 2-9 years
D, 2-year samples of fungal community and height SDS at 2-9 years
Samples are ordered by increasing height SDS and BMI SDS. The heat maps describe the overall patterns across anthropometric measurements, and the total abundance score is 1.


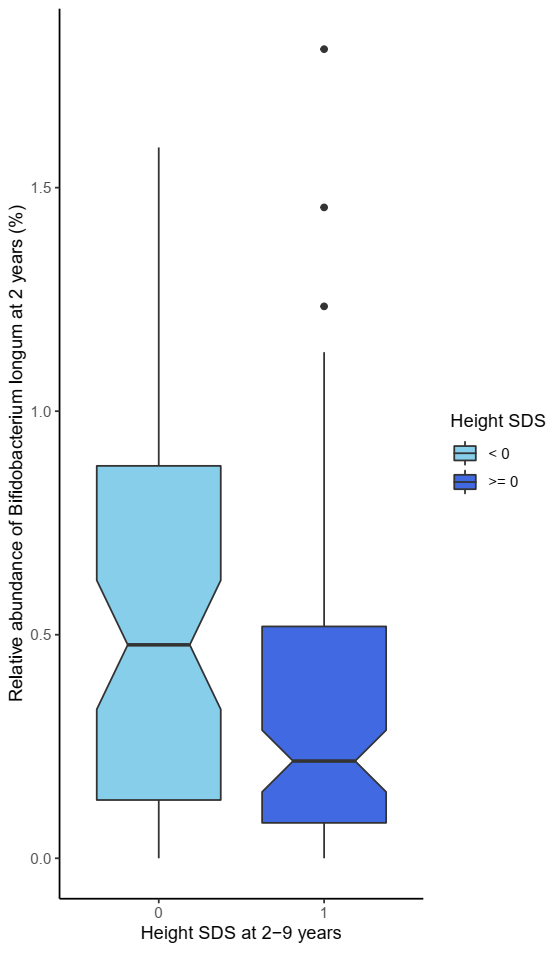
**Supplementary Figure 3. Height SDS at 2-9 years and relative abundance of Bifidobacterium longum at 2 years.**The horizontal lines indicate the lower, median and upper quartile. Whiskers extend 1.5 times the interquartile range from the upper and lower quartile.

**Standard Curve Calculations of Bacterial and Fungal rRNA genes**

The concentrations used are the concentrations from the faecal sample tubes, which are about 20 times diluted compared to the faecal samples themselves due to dilution in the Cary-Blair Transport Medium.

**Concentration calculation of bacterial DNA**To convert the CT values into 16S DNA concentration, we created a positive control of known bacterial concentration. The positive control was collected by purifying DNA from a cultured *Escherichia coli* strain. 50 µL of E. coli DNA was diluted with 50 µL of nuclease-free water. We used Qubit (Thermo Fisher Scientific) to calculate the DNA concentration in the sample.

*DNA concentration in positive control*Concentration in *E. coli* sample: 6.65 ng/µL (Qubit measurement)

First control (10^-1^): 0.0665 ng/µL
Second control (10^-2^): 0.0665 ng/µL
Third control (10^-3^): 0.00665 ng/µL

*V3-V4 copy number in 1 mL of* E. coli *(positive control)*Total length of V3-V4 region (between PRK341F and PRK806R) = 466 bp (<https://doi.org/10.1002/bit.20347>). Total basepair length of E. coli strain K-12 substrain DH5-alpha = approx. 4.6 Mbp (type str. K-12, substr MG1655 4.64, and closely related NEB5-alpha has 4.58 Mbp). (<https://www.ncbi.nlm.nih.gov/pmc/articles/PMC5105096/> and <https://www.ncbi.nlm.nih.gov/genome/?term=Escherichia+coli>)

Mass of E. coli genome:

$$m=n\times1.096\times\frac{{10}^{-21}g}{bp}$$

$$m=4.5 bp\times{10}^{6}\times1.096\times\frac{{10}^{-21}g}{bp}$$

$$m=4.932\times{10}^{-15} g=4.932 fg (weight of total E. coli genome)$$

There are 7 copies of the 16S rRNA gene region in the *E. coli* genome. 1 genome of 4.932 x 10^-15 thus contains seven copies of 16S.DNA concentration in positive control: 6.65 ng DNA /µL. Mass of *E. coli* genome containing 7 16S rRNA gene regions = 4.932 x 10^-15 g

$$C_{DNA}=6.65\frac{ng}{\mu L}=6.65\times\left( \frac{{10}^{-9}g}{{10}^{-6}L} \right)|\times(\frac{{10}^{3}}{{10}^{3}})$$

$$C_{DNA}=6.65\times\frac{{10}^{-6}g}{mL}$$

*E. coli and 16S concentration in positive control.*

$$C_{E. coli}=\frac{6.65\times{10}^{-6}\frac{g}{mL}}{4.932\times{10}^{-15}g}=1.348448\times{10}^{9}\frac{\left( E. coli \right)}{mL}$$

$$C_{16S}=7\frac{16S}{E. coli}\times1.348448 x {10}^{9}\frac{E. coli}{mL}=9.438362\times{10}^{9}\frac{16S}{mL}$$

16S concentration in first positive control: 9.44 x 10^8 16S/mL = 8.975 log (16S conc.)
16S concentration in second positive control: 9.44 x 10^7 16S/mL = 7.975 log (16S conc.)
16S concentration in third positive control: 9.44 x 10^6 16S/mL = 6.975 log (16S conc.)

| **16S standard curve calculation** | | | | | | | | | | | | | | | | | | |
| --- | --- | --- | --- | --- | --- | --- | --- | --- | --- | --- | --- | --- | --- | --- | --- | --- | --- | --- |
| Plate number | 1 | 2 | 3 | 4 | 5 | 6 | 7 | 8 | 9 | 10 | 11 | 12 | 13 | 14 | 15 | 16 | 17 | Mean CT value |
| 10^-1 | 18.53 | 18.28 | ^a^ | 18.67 | 18.72 | 19.36 | 18.99 | 19.03 | 18.98 | 19.10 | 19.07 | 19.13 | 19.12 | 19.16 | 19.32 | 19.16 | 19.25 | 18.99 |
| 10^-2 | 22.08 | 22.04 | 22.67 | 22.13 | ^b^ | 22.63 | 22.54 | 22.57 | 22.45 | 22.70 | 22.66 | 22.82 | 22.85 | 22.76 | 22.73 | 22.45 | 22.72 | 22.55 |
| 10^-3 | 25.51 | 25.39 | 26.35 | 25.48 | 26.94 | 26.15 | 26.15 | 26.27 | 26.06 | 26.34 | 26.07 | 26.35 | 26.40 | 26.18 | 26.40 | 26.08 | 26.44 | 26.15 |

^a^ Not registered
^b^ Removed due to probable mixing of samples

|  | **Mean CT value** | **Log(16S conc)** |
| --- | --- | --- |
| **1st positive control** | 18.994 | 8.974972 |
| **2nd positive control** | 22.551 | 7.974972 |
| **3rd positive control** | 26.152 | 6.974972 |

To the left is the linear regression line calculated from 3 different dilutions of the positive control on all 17 PCR plates. Based on the regression line, the following equation was used to calculate the 16S copy number:

$$Log conc 16S \left( CT \right)=-0.2794 CT+14.279$$

**Concentration calculation of fungal DNA**To convert the CT values into ITS DNA concentration, we created a positive control of known fungal concentration that was integrated in the analyses. The positive control was made by carefully dissolving a commercially produced fresh baking yeast block consisting of a *S. cerevisiae* strain into sterile water, and we then extracted the DNA in the same manner as the DNA from the faecal samples were extracted. Then, we quantified the DNA concentration of the positive control with Qubit. We next calculated the concentration of ITS copies in the positive control, as shown below. Then, we made three dilutions of the positive control, which we included in all 17 PCR plates. We quantified the CT values of these dilutions with the qPCR in the same way as the faecal sample CT values were quantified (see the method section), and thereafter used the average of the CT values to make a linear standard curve. Since the positive controls were diluted by tenths, we used the logarithmic function of the ITS concentration to obtain a linear standard curve. This standard curve made it possible to convert CT values to ITS concentration in the other samples.

*DNA concentration in positive control*
Concentration in positive control of *Saccharomyces cerevisiae* with Qubit: 0.102 µg/mL.

*ITS copy number in 1 mL of* S. cerevisiae *(positive control)*
Total ITS length: 500-600 bp.
ITS1F-ITS2: 314 bp (http://www.ncbi.nlm.nih.gov/pmc/articles/PMC4059633/)
*Saccharomyces cerevisiae* total genome: 12 157 105 bp (haploid) (<http://www.yeastgenome.org/strain/S288C/overview>)

$$Mass of S. cerevisiae genome=$$

$$m=n\times1.096\times\frac{{10}^{-21}g}{bp}$$

$$m=12157105 bp\times1.096\times\frac{{10}^{-21}g}{bp}$$

$$m=1.332418708\times{10}^{-14}g$$

$$m=0.013324 pg$$

It is uncertain how many tandem loops there are in the fungal genome. If there were one copy number of ITS1 in a haploid genome, 0.0133 pg of fungal DNA contains one copy of ITS1.

Concentration in positive control: 0.102 µg/mL = 102 ng/mL = 102 000 pg/mL.

$$Mass of S. cerevisiae genome containing one copy=$$

$$m_{one}= 0.013324\frac{pg}{copy}$$

$$ITS copy concentration in positive control=$$

$$C_{ITS}=\frac{102000\frac{pg DNA}{mL}}{0.013324\frac{pg DNA}{copy}}=7655251.265\frac{ITS copies}{mL}$$

$$C_{ITS}=7 655 251\frac{ITS copies}{mL}$$

$${10}^{-3}dilution: 7655 \frac{ITS copies}{mL}$$

$${10}^{-4}dilution: 765.5 \frac{ITS copies}{mL}$$

$${10}^{-5}dilution: 76.55 \frac{ITS copies}{mL}$$

**ITS standard curve**Mean CT values for positive controls in all 17 plates were calculated. From the above calculated ITS concentrations in the three dilutions of the positive control, we constructed a normal curve based on ITS concentration (logarithmically expressed for normality) and CT values.

| Dilutions | ITS/mL (x) | log(x) | Mean CT values (y) |
| --- | --- | --- | --- |
| 10^-3^ | 7655 | 3.883945195 | 28.13 |
| 10^-4^ | 765.5 | 2.883945195 | 31.48 |
| 10^-5^ | 76.55 | 1.883945195 | 35.69 |

To the right is the linear regression line calculated from 3 different dilutions of the positive control on all 17 PCR plates. Based on the regression line, the following equation was used to calculate the ITS copy number:

$$\log\left( \frac{ITS}{ml} \right)=\frac{42.664-CT value}{3.7787}$$

In the calculations following in the article, the natural logarithm (of *e*) was used.

1. Avershina E, Lundgard K, Sekelja M, et al. Transition from infant- to adult-like gut microbiota. *Environ Microbiol.* 2016.

2. Yu Y, Lee C, Kim J, Hwang S. Group-specific primer and probe sets to detect methanogenic communities using quantitative real-time polymerase chain reaction. *Biotechnology and bioengineering.* 2005;89(6):670-679.

3. Tang J, Iliev ID, Brown J, Underhill DM, Funari VA. Mycobiome: Approaches to analysis of intestinal fungi. *Journal of immunological methods.* 2015;421:112-121.

4. Edwards JE, Forster RJ, Callaghan TM, et al. PCR and Omics Based Techniques to Study the Diversity, Ecology and Biology of Anaerobic Fungi: Insights, Challenges and Opportunities. *Frontiers in microbiology.* 2017;8:1657.

5. Taylor DL, Walters WA, Lennon NJ, et al. Accurate Estimation of Fungal Diversity and Abundance through Improved Lineage-Specific Primers Optimized for Illumina Amplicon Sequencing. *Applied and environmental microbiology.* 2016;82(24):7217-7226.

6. Schei K, Avershina E, Øien T, et al. Early gut mycobiota and mother-offspring transfer. *Microbiome.* 2017;5(1):107.

7. Edgar RC. UPARSE: highly accurate OTU sequences from microbial amplicon reads. *Nat Meth.* 2013;10.
